# *bAIcis*: A Novel Bayesian Network Structural Learning Algorithm and Its Comprehensive Performance Evaluation Against Open-Source Software

**DOI:** 10.1089/cmb.2019.0210

**Published:** 2020-05-07

**Authors:** Lixia Zhang, Leonardo O. Rodrigues, Niven R. Narain, Viatcheslav R. Akmaev

**Affiliations:** BERG Health, Framingham, Massachusetts, USA.

**Keywords:** Bayesian network, causal inference, structural learning

## Abstract

Structural learning of Bayesian networks (BNs) from observational data has gained increasing applied use and attention from various scientific and industrial areas. The mathematical theory of BNs and their optimization is well developed. Although there are several open-source BN learners in the public domain, none of them are able to handle both small and large feature space data and recover network structures with acceptable accuracy. *bAIcis*^®^ is a novel BN learning and simulation software from BERG. It was developed with the goal of learning BNs from “Big Data” in health care, often exceeding hundreds of thousands features when research is conducted in genomics or multi-omics. This article provides a comprehensive performance evaluation of *bAIcis* and its comparison with the open-source BN learners. The study investigated synthetic datasets of discrete, continuous, and mixed data in small and large feature space, respectively. The results demonstrated that *bAIcis* outperformed the publicly available algorithms in structure recovery precision in almost all of the evaluated settings, achieving the true positive rates of 0.9 and precision of 0.8. In addition, *bAIcis* supports all data types, including continuous, discrete, and mixed variables. It is effectively parallelized on a distributed system and can work with datasets of thousands of features that are infeasible for any of the publicly available tools with a desired level of recovery accuracy.

## 1. Introduction

Causal inference, the process of finding relationships that describe cause-and-effect events, involves inferring the consequences in a counterfactual reality where an alternative potential cause occurred (Pearl, 2010; Morgan and Winship, 2014). As Pearl pointed out, causal and statistical inferences have fundamental differences since they focus on causation and association, respectively (Pearl, 2009a). Moreover, when compared with statistical inference, causation requires one step further to investigate the outcomes by changing their conditions. Identifying causal relationships generally requires three levels of empirical evidence: temporal precedence, empirical association, and nonspurious relationships (Chambliss and Schutt, 2018). One traditional approach for testing causal hypotheses is to conduct a well-designed experiment, where it is possible to control and intervene the condition, monitor the outcome change, and finally reach the causal conclusion. A clinical trial is a typical example that aims at demonstrating that one drug is the cause of improved outcomes. However, in certain scientific fields, such as epidemiology and social science, most studies are, by nature, observational rather than experimental (Rothman et al., 2008); in addition, in new domains such as climate research (Von Storch, 1999) and microarray measurements of gene expression (Nelson et al., 2004), where the number of measured variables can be up to tens of thousands, even when experimental interventions are available, performing such a number of experiments is costly, time-consuming and takes extensive resources.

Aiming at detecting causal relationships in observational data, Pearl debated that genuine causal inferences are possible from passive observations, introduced a minimal-model semantics of causation, and developed the Inductive Causation algorithm to identify causal relations rather than spurious covariations (Pearl and Verma, 1995). Moreover, the theory of causal transportability discussed that causal relations learned from experiments can be transferred to a different environment where only observational data are available (Pearl and Bareinboim, 2011). Chickering discussed learning causation structure by a scoring metric and advantages taken from score-equivalent evaluation criterion in identifying high-scoring structures (Chickering, 1996, 2002). These altogether led to the development of graphical causal modeling, a methodology widely used to describe the conditional independence relationships among a set of random variables based on the probability theory (Pearl, 2009b).

In a graphical model, nodes represent variables of interest, edges connecting nodes represent dependencies among the variables, and arrows, if they exist, refer to directionalities of the dependencies, for example, causal relationship. Bayesian networks (BNs) (Pearl, 2011) are a specific type of graphical models that are directed acyclic graphs (DAGs), thus all of the edges are directed with no cycles existing in the model. As a marriage of causality and probability theories, BNs convey knowledge of data-generating process and are capable of identifying and inferring causation in both experimental and observational data. In this regard, BNs received a great amount of attention from various scientific fields such as reverse engineering of gene regulatory network (Baldi and Long, 2001; Hartemink et al., 2001; Xiao et al., 2015) and explanations of social phenomena (Whitney et al., 2011; Farasat et al., 2015).

In recent years, many algorithms have been developed for learning causal relationships among a set of variables under the BN framework. *Rimbanet* is a software package focusing on reconstructing integrative molecular BNs to understand biological systems (Zhu et al., 2004); *deal* is an R package that provides algorithms for analyzing data by using BNs restricted to conditionally Gaussian networks (Boettcher and Dethlefsen, 2003); *bnFinder*, scripted in Python, implements an exact learning algorithm for BNs reconstruction with parallel computing for multicore and distributed systems (Frolova and Wilczyński, 2018); and *sparsebn*, an R package, is designed to deal with large feature space data (Aragam et al., 2017). Most of these algorithms are only able to handle either small or large feature space data effectively, with only a few being able to deal with both regimes effectively.

In this article, we introduce *bAIcis*^®^, a BN structure learning algorithm developed and implemented by BERG LLC. It was developed with the goal of learning BNs from “Big Data” in health care, which often exceeds hundreds of thousands features when the research is conducted in genomics or multi-omics. Thus, the algorithm is capable of handling data in both small and large feature spaces effectively, and it has built-in capability to run in both multicore desktops and distributed systems, making the algorithm efficient for datasets in any scale. The purpose of this article is to benchmark the statistical performance of *bAIcis* and a number of open-source BN learners with regard to the accuracy of network recovery, scalability, and computation time.

## 2. Methods

### 2.1. Bayesian networks

**FIG. 1.**
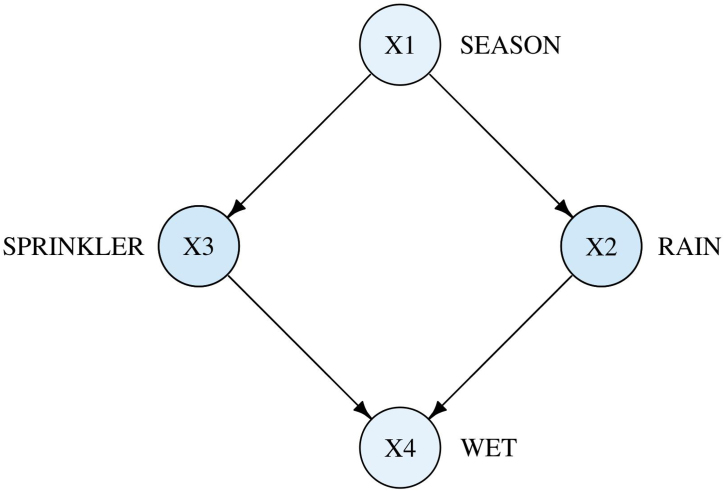
A BN representing the causal relationships among four variables: the season of the year (*X_1_*), whether rain falls (*X_2_*), whether the sprinkler is on (*X_3_*), and whether the grass gets wet (*X_4_*). BN, Bayesian network.

BNs, which encode the conditional independencies among a set of variables in a DAG, are usually used as a presentation of cause-effect relationships (Pearl, 2011). Each directed edge indicates a direct causal relationship, whereas the absence of an edge refers to no direct causal impact. Hence, it is easy to borrow kinship relation terms to describe the relationships in a graph, such as parent, child, ancestor, and descendent. For example, an arrow *X* → *Y* refers to *X* as a parent of *Y* and *Y* as a child of *X* (Fig. 1). As described in Figure 1, both sprinkler and rain can directly influence whether the grass is wet; whereas the influence of seasonal variations on the wetness of grass is mediated by other conditions. Further, if it is raining, the grass is wet regardless of the season condition. This statement satisfies the Markov condition, which states that every node in a BN is conditionally independent of its nondescendent nodes, given its parent nodes. In this example, the joint distribution of all four variables can be factorized by this BN as

$$P\left(X_{1},X_{2},X_{3},X_{4}\right)=P\left(X_{1}\right)P\left(X_{2}|X_{1}\right)P\left(X_{3}|X_{1}\right)P\left(X_{4}|X_{2},X_{3}\right).$$

In general, given nodes **X** = (*X*_1_, *X*_2_…, *X_n_*), the joint probability function for any BN is

$$P\left(X\right)=\prod_{i=1}^{n}P\left(X_{i}|parents\left(X_{i}\right)\right).$$

Hence, a BN factorizes a global full joint distribution of all variables to a set of local conditional distributions for each variable given its parents depending on the model structure.

### 2.2. Bayesian network tools and packages

The prevalent techniques of learning BNs can be grouped into two broad categories: score-based algorithms and constraint-based algorithms (Yu et al., 2016). Score-based algorithms assign a score to each candidate BN on measuring goodness of fit and attempt to return a causal structure that maximizes the score, for example, Bayesian information criterion (BIC) (Chickering, 2002; Tsamardinos et al., 2006; Carvalho, 2009); whereas constraint-based algorithms learn the BN structure based on Markov condition by a series of local conditional independence constraints and construct a graph that meets the independent relationships (Spirtes and Glymour, 1991; Pearl and Verma, 1995; Claassen and Heskes, 2012). The advantages and disadvantages of the two algorithms were discussed elsewhere (Spirtes, 2010; Triantafillou and Tsamardinos, 2016; Scutari et al., 2018).

*bAIcis* is a score-based BN learning algorithm with a BIC score criterion. *bAIcis* is a proprietary model-search algorithm that learns the network structure from the data by maximizing the BIC score in two phases. In the first phase, *bAIcis* generates optimal combinations of parents for each individual node by local BIC, and in the latter phase *bAIcis* incorporates those families to construct a final optimal network by global BIC. Bayesian methods are applied by using prior distributions to estimate the parameters (Heckerman, 1998).

There are several BN learners scripted in either R or Python available in the public domain. The tools *bnFinder* (Frolova and Wilczyński, 2018), *bnlearn* (Scutari, 2010), *deal* (Scutari, 2010), *pcalg* (Kalisch et al., 2012), *Rimbanet* (Zhu et al., 2004), and *sparsebn* (Aragam et al., 2017) were selected to benchmark the causal structure recovery.

Table 1 displays a summary of these BN tools, including *bAIcis*, in regard to network type, structure learning, and implemented learning algorithm. The *Network Type* section represents the ability of the method to work with continuous, discrete, or mixed variables; the *Structure Learning* section provides the level of flexibility of the obtained network solution, where weighted edges indicate the strength of dependencies connecting two nodes and enable the flexibility to scale down or up the networks; and *Learning Algorithm* indicates whether the tool is score-based or constraint-based. The majority of the selected tools implemented score-based learning algorithms and are consistent with *bAIcis*, except the R package *pcalg* where a PC algorithm is used for comparison. In the R package *bnlearn*, which implements both algorithms, the score-based hill-climbing greedy search algorithm is utilized.

**Table 1.** Summary of the Evaluated Bayesian Network Tools, Along with the Capability of Dealing with Different Data Types in the Network, the Solution Format from Network Structure Learning, and the Implemented Learning Algorithm

	Network type			Structure learning solution		Learning algorithm	
	Continuous	Discrete	Mixed	One solution	Weighted edges	Score based	Constraint based
bAIcis^®^	✓	✓	✓	✓	✓	✓	✗
Rimbanet	✓	✓	✓	✓	✗	✓	✗
bnlearn	✓	✓	✓	✓	✗	✓	✓
deal	✗	✓	✓	✓	✗	✓	✗
sparsebn	✓	✓	✗	✓	✓	✓	✗
pcalg	✓	✓	✗	✓	✗	✗	✓
bnFinder	✓	✓	✓	✓	✗	✓	✗

## 3. Benchmarking Study

### 3.1. Synthetic networks

A set of synthetic networks and datasets were generated to evaluate and compare the performance of the BN learners in both small and large feature spaces, where the number of nodes is below 50 and beyond 500, respectively. Since structural equation models (SEMs) are considered as a language for causality (Wright, 1921; Pearl, 2013), linear SEMs is utilized to simulate the synthetic data for network reconstruction.

Networks were generated by predefining the network type, topology structure, node size, and sample size. Network type represents the data type for nodes, which could be continuous, discrete, and mixed networks (mixed with continuous and discrete nodes with a condition only allowing discrete variables to be the parents of discrete child nodes). Topology structure, the overall nodes’ degree distribution, is predefined as either random network or scale-free network. In the discrete-only networks, the number of parents for a discrete child has to be constrained, otherwise the discrete level of the child would inflate. Thus, scale-free topology was only implemented in continuous networks. Node size constrains the number of involved variables, and sample size sets contain the number of observations.

A total of 36 datasets were produced for the experimental design configurations. Under each setting, generalized linear SEMs were utilized to generate synthetic data as follows:

- A continuous child *X_i_* with all continuous parents was simulated from a Gaussian distribution $X_{i}∼N\left(\sum_{j\in \phi_{i}}\beta_{ji}X_{j},\sigma_{i}^{2}\right)$, where $\phi_{i}$ refers to a set of parents of node *X_i_*, *β_ji_* is the structural parameter associated with parent *X_j_* generated from *N*(2, 0.8) with the sign (positive or negative regulation) simulated from a binomial distribution with probability 0.5, and $\sigma_{i}^{2}$ is the error term generated from *N*(1, 0.01);
- A discrete child was simulated by a multinomial distribution, ensuring conditional dependencies between each parent and the child;
- A continuous child with discrete or mixed parents was simulated from a mixture Gaussian distribution.

Since some BN tools learn network structures by the order of variables presented in datasets, for example, *bnlearn*, the order was shuffled to eliminate the impact. In addition, 20 replications were conducted for each configuration to capture the variation. The open-source BN learners were run with their default settings. Regarding *bnFinder*, the search space for each node was limited to six parent nodes to expedite algorithm running.

In addition to benchmarking performance in small feature space with node size at 10, 20, and 50, a study in large feature space was also conducted, where *bnlearn* and *sparsebn* were selected since both claim to be efficient for data in large feature space.

### 3.2. Network learning evaluation

Performance was evaluated and benchmarked across the selected BN tools, regarding structure learning accuracy and computation time. Structure learning was evaluated on three metrics: true positive rate (TPR), precision, and false positive rate (FPR). TPR, also called as recall, measures the capability of detecting a true edge; precision, true discovery rate, is the ratio of true edges among all detected ones; and FPR refers to the type I error, that is, the ratio falsely detected edges among all nondirected relations. Two-sided paired sample *t*-test was assessed to compare *bAIcis* with all the other BN learners on the three metrics. Regarding running time, for small feature space, the number of running CPU was restricted to be 1 for all algorithms, except *bnFinder*, which was allocated a total of 32 CPUs.

## 4. Results

### 4.1. Small feature space

#### 4.1.1. Structure learning

**FIG. 2.**
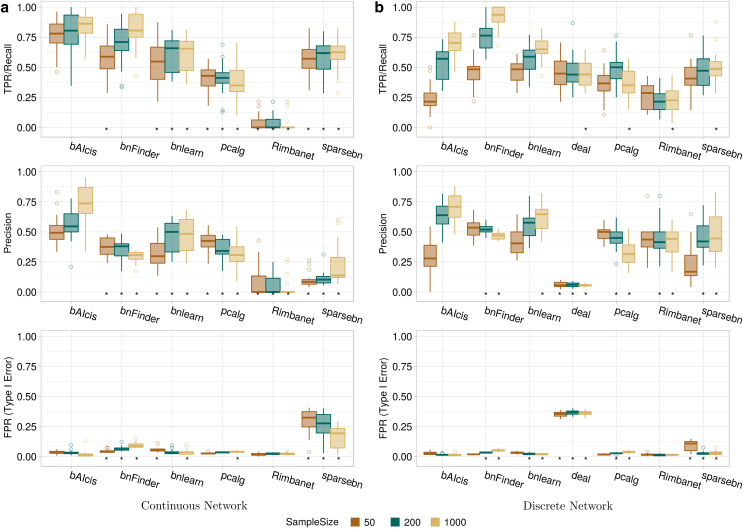
Evaluation and comparison in metrics of edge detection among BN tools for synthetic networks of random topology with 20 nodes across different sample sizes in continuous network (left) and discrete network (right). Each panel comprises three metrics plots for TPR, precision, and FPR shown from top to bottom. In each metric plot, *y*-axis presents the metric value ranging from 0 to 1; *x*-axis shows the compared BN tools; and the distributions of metric rate across 20 replicates are summarized in boxplots stratified by sample size. An asterisk below a boxplot indicates that *bAIcis*^®^ performs statistically significantly better than the corresponding tool from a two-sided paired sample *t*-test at significant level 0.5. FPR, false positive rate; TPR, true positive rate.

Networks with 50, 200, and 1000 observations were generated, submitted to the BN learners’ analysis, and evaluated for TPR, precision, and FPR. In the continuous network with 20 nodes (Fig. 2a), *deal* was not evaluated due to its inability of handling continuous networks. Regarding TPR, all analyzed BN learners, except *Rimbanet*, were able to recover a comparable number of true edges, even in the 50-observations networks. However, *bAIcis* was able to significantly recover more true edges, compared with most BN learners. For FPR and sample size of 50 and 200, all the values were dense within a relatively low range, except *sparsebn* with a median-FPR higher than 0.25. In the precision, *bAIcis* outperformed all BN learners across all observation sizes and the superiority was statistically significant at a level of 0.05 by the two-sided paired sample *t*-test. As sample size increased to 1000, *bAIcis* identified less false edges in the learned network structure with increasing precision and decreasing FPR; whereas *bnFinder* and *pcalg* showed an opposite trend. *Rimbanet* was less impacted by sample size, with a comparatively flat trend in all three metrics. We evaluated the same metrics in the discrete network (Fig. 2b).

The difference in performance was slightly less dramatic compared with the continuous network. *Rimbanet* overall showed a better performance in the discrete network. *bAIcis* was not top ranked in TPR and precision under the 50-observations scenario; however, it shone and outperformed the competitors in precision when sample size increased to 200 and beyond. In the metric of TPR, overall *bnFinder* performed the best whereas *Rimbanet* was left behind; regarding precision and FPR, all values were relatively close, except *deal* whose values were below 0.2 and above 0.3, respectively. The trends by the sample size shared the pattern with the continuous network. In addition to *Rimbanet*, *deal* presented as another member in the unimpacted group.

The results of all 36 analyzed settings can be seen in Figure 4a–c. Each figure exhibits one evaluation metric and is summarized in a plot integrating the outcomes throughout all 36 settings. Nonsupported outcomes were left blank in figures, for example, *deal* in continuous networks. Besides, the results for *deal* and *bnFinder* with 50 nodes were not applicable because *deal* failed to learn a network with 50 nodes due to its memory limit, and *bnFinder* required an unexpectedly long computation time (more than 1 day on 32 CPUs). For all three metrics, the figure patterns preserve the same, with slight differences across different node sizes given the network type and topology structure. Overall, the boxplots got compressed as the node size increased, indicating that the variance among replicates was reduced. The performance of *bAIcis* was quite stable and not largely affected by the node size. Comparing figures vertically, we can see that patterns change mostly due to the network type rather than topology structure, although the average performance goes down on a small scale in scale-free topology. *bAIcis* still emerges as the winner in the mixed network for all three metrics.

#### 4.1.2. Computation time

Computation time was calculated by using the mean and standard deviation across 20 replications as summarized statistics (Table 2 and Fig. 4d). No dramatic difference was observed in running time between continuous and discrete networks when sample size was 50 and 200, except *bnFinder* whose running time was considerably reduced in handling discrete networks (Table 2). Among all, *bnlearn* and *pcalg* held the fastest completion times that were in milliseconds; whereas *bnFinder* and *deal* were the most time-consuming algorithms. As expected, the computation cost was higher as the sample size or the node number increased. The increase varied depending on the different algorithms and network types (Fig. 4d). *Rimbanet* and *bnlearn* were unaffected by the sample size and both display a plateau in each subfigure of Figure 4d. Regarding *bAIcis*, it took from 30 seconds to learn a 50-samples network to 5 minutes to learn a 1000-samples network, with 20 nodes.

**Table 2.** Summary for Computation Time (in Seconds) Among Bayesian Network Tools for Synthetic Networks of Random Topology Structure with 20 Nodes in Continuous and Discrete Networks

Sample size**Bayesian tool	Network type					
	Continuous			Discrete		
	50	200	1000	50	200	1000
bAIcis	34.27 (3.839)	44.72 14.856	61.07 (30.979)	35.4 (7.674)	48.26 (8.208)	319.14 (61.843)
bnFinder	8326.7 (1223.209)	30891.56 (2269.565)	158702.03 (10325.297)	248.1 (44.935)	767.12 (131.194)	3213.06 (465.16)
bnlearn	0.02 (0.006)	0.03 (0.012)	0.06 (0.022)	0.02 (0.006)	0.02 (0.005)	0.03 (0.01)
deal	NA	NA	NA	4616.59 (1074.237)	5747.56 (1511.478)	5644.55 (1339.394)
pcalg	0.02 (0.005)	0.02 (0.008)	0.03 (0.018)	0.05 (0.007)	0.11 (0.03)	0.34 (0.169)
Rimbanet	9.4 (1.845)	9.89 (1.367)	10.85 (1.583)	11.29 (0.947)	11.22 (0.776)	12.82 (0.578)
sparsebn	3.37 (0.258)	3.33 (0.197)	3.57 (0.551)	3.04 (0.304)	7.12 (0.925)	30.88 (4.102)

The computation time is displayed as mean (stardard deviation) across 20 replicates.

### 4.2. Large feature space

**FIG. 3.**
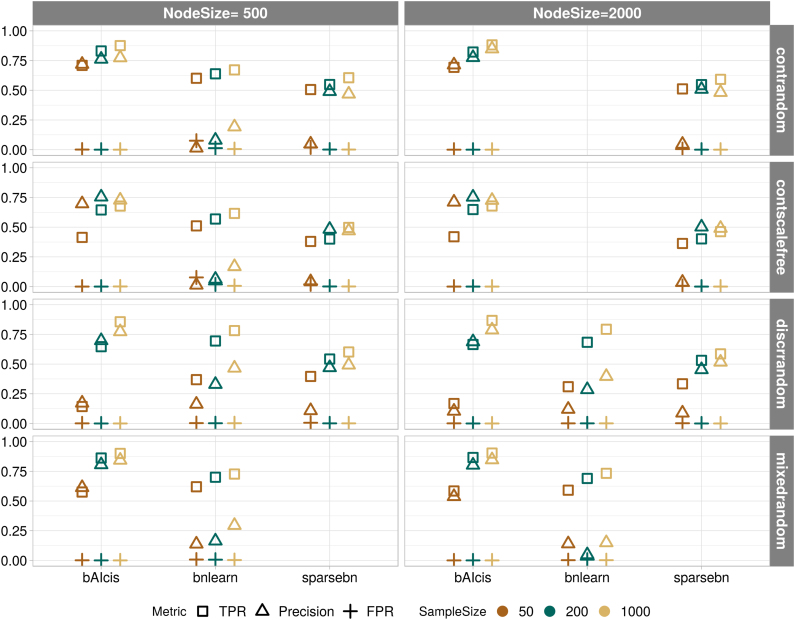
Median points of TPR (in square), precision (in triangle), and FPR (in cross) under large feature space data for three BN leaners: *bAIcis*, *bnlearn*, and *sparsebn*. It is an integrated figure composed of eight subfigures stratified by network type and topology structure (continuous random topology network, continuous scale-free topology network, discrete random topology network, and mixed random topology network) vertically and node size (500 and 2000) horizontally; in each subfigure, *y*-axis presents the metric value ranging from 0 to 1, *x*-axis shows the compared BN tools, and median points are further stratified by sample size (50 in brown, 200 in green and 1000 in yellow). The variance among the replicates was extremely small and hence was not shown. The outcomes for *bnlearn* on the continuous networks with 2000 nodes are left blank due to its unexpected long running time.

The benchmarking study under large feature space with a node size at 500 and 2000 was conducted on *bAIcis*, *bnlearn*, and *sparsebn*. Figure 3 displays the integrated plot for metrics (in median) stratified by network type, topology structure, node number, and sample size. Because of the extremely large base of nonconnected relations, the FPR values for the three algorithms mostly all reached the bottom. Comparing algorithms on the other two metrics, *bAIcis* recovered more true edges and less false edges when compared with *bnlearn* and *sparsebn*; *bnlearn* failed to persist the good performance in large feature space; whereas *sparsebn* climbed steadily as more nodes were involved in the network. Computation time wise, *bAIcis* enabled its parallel functionality by running on distributed systems, and it was able to finish the network learning within 45 minutes; whereas the other two were running on one single CPU, and *sparsebn* turned to be more efficient than *bnlearn* on average. It took *bnlearn* more than 1 day to tackle the continuous networks with 2000 nodes, and thus the corresponding outcomes were left blank.

## 5. Discussion

**FIG. 4.**
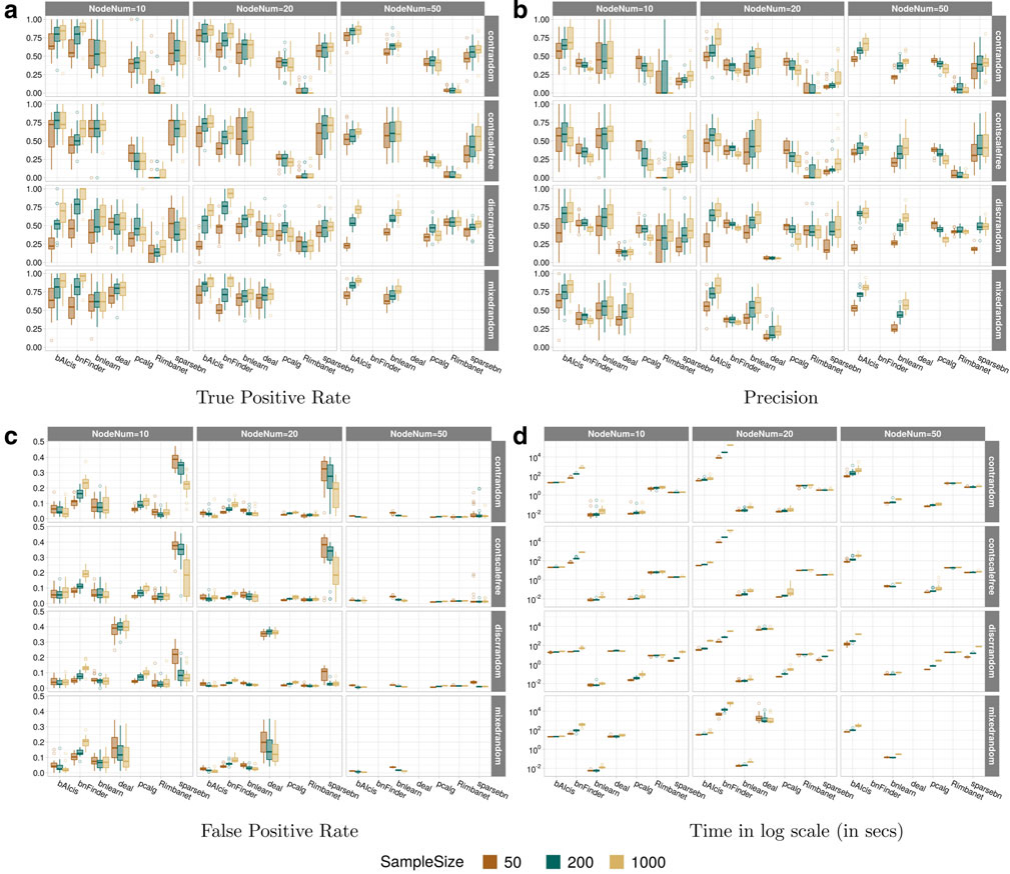
Boxplots of metrics under small feature space data for all BN learners. Panels represent the outputs from TPR, precision, FPR, and computation time in log scale (in seconds). Each panel is an integrated figure composed of 12 subfigures stratified by network type and topology structure (continuous random topology network, continuous scalefree topology network, discrete random topology network, and mixed random topology network) vertically and node size (10, 20 and 50) horizontally; in each subfigure, boxplots are further stratified by sample size (50 in brown, 200 in green, and 1000 in yellow).

We have conducted a benchmark study to evaluate and compare the performance of the BN learners to recover causal structure under synthetic data against various settings of network types, topology structures, numbers of nodes, and sample size. *bAIcis* was capable of handling networks in both small and large feature regimes effectively and supported discrete, continuous, and mixed networks. Further, the complexity of the network structure impacted *bAIcis* little as it had stable performance in the accuracy of structure recovery (Fig. 4). Although the performance in scale-free structure was slightly lower, it is mostly due to the unexpectedly large number of parents for certain nodes.

The majority of the BN algorithms, including *bAIcis*, gained power to identify edge connections without sacrificing precision as the sample size increased, whereas *bnFinder* and *pcalg* failed to differentiate between true and false connections when more observations were available, as observed with a decrease in precision and an increase in FPR.

*bAIcis* performed superior to the other open-source BN tools under the majority of simulation settings by identifying directed edges with high TPR and precision and low FPR values. The superiority was stronger in large feature spaces. Among three metrics, *bAIcis* outperformed the most in precision, which is usually considered as the major feature in real-life applications where the ground truth is unknown. High precision achieved by *bAIcis* indicates that the networks recovered by it are of high fidelity and have meaningful links between the variables.

Algorithm computation time in both *bnlearn* and *pcalg* was quite superior to the others, whereas the running time for *bnFinder* gained dramatically as the node size increased. Although *bAIcis* was not the fastest algorithm in running time for small networks, the time spent, less than 5 minutes, is still considered as reasonable and acceptable. If the *bAIcis* parallelization functionality is enabled, the software can run on a distributed system and, hence, the spent time would be dramatically reduced.

Although not evaluated in this study, *bAIcis* possesses several other differentiating features that enlarge its real application and give the flexibility on the postnetwork analysis and network representation. For example, *bAIcis* provides comprehensive output containing the estimated parameters of all predicted dependencies. The edge matrix can be easily converted to a topologic graph. Moreover, *bAIcis* can be run from an R-library wrapper that automates the run as well as post-BN data processing, for example, network visualization in Cystoscape or igraph.

This benchmarking study gives a comprehensive evaluation on the performance of BN structure learners and demonstrates the advantages of *bAIcis* compared with open-source BN learners. Since the synthetic data were generated by linear SEMs, it is hard to generalize the results on nonlinear relationships. But based on the study results, we can conclude that *bAIcis* (1) supports continuous, discrete, and mixed networks and it performs stably and effectively for both small and large feature space; (2) achieves high precision; and (3) is implemented with parallel-computing capability that allows it to run in both multicore desktops and distributed systems.

## 6. Conclusion

BNs provide an alternative paradigm for statistical learning where causal relationships connecting various data features may open up possibilities for either hypothetical or real-life interventions into the studied system. BNs also allow for evidential reasoning by simulation or propagation through the network connections. BNs have wide real-world application across a variety of scientific and industrial areas, for example, learning gene regulation in life sciences, understanding most optimal strategies in patient care, and identifying root causes of poor clinical outcomes and phenotypes. From the benchmarking study, we can conclude that *bAIcis* is one of the most accurate BN structural learners compared with a number of publicly available algorithms. Moreover, the *bAIcis* software is effectively parallelized on a distributed system and hence can manage extremely large datasets that are currently common in life sciences, including genomics and multi-omics data.
